# P41 Spotting the needle amongst the haystack - when asthma isn’t asthma

**DOI:** 10.1093/rap/rkac067.041

**Published:** 2022-09-28

**Authors:** Ruqaiya Al Jashmi, Kathy Gallagher, Despina Eleftheriou, Elena Moraitis

**Affiliations:** Great Ormond Street Hospital, Department of Rheumatology, London, United Kingdom; Great Ormond Street Hospital, Department of Rheumatology, London, United Kingdom; Great Ormond Street Hospital, Department of Rheumatology, London, United Kingdom; Great Ormond Street Hospital, Department of Rheumatology, London, United Kingdom

## Abstract

**Introduction/Background:**

Eosinophilic granulomatosis with polyangiitis (EGPA), previously known as Churg-Strauss Syndrome, is a rare, small to medium vessel ANCA associated vasculitis. Hallmarks of EGPA include asthma, chronic rhinosinusitis, and peripheral neuropathy.

EGPA is characterized by a prodrome of asthma and allergic rhinitis, followed by peripheral blood hyper-eosinophilia and accumulation of extravascular eosinophils, and finally systemic vasculitis. Extra-pulmonary involvement is common, sometimes with fatal outcomes. The onset of EPGA is typically between 25-50 years; however, EGPA also occurs during childhood and has a significant morbidity and mortality.

**Description/Method:**

Our patient presented to the emergency department with a 2-week history of lethargy, wheeze and left sided neck swelling.

After testing COVID-19 positive eight months prior to this, she developed wheezy episodes and was subsequently diagnosed with asthma which was managed with bronchodilators as required. She was reviewed by an allergist who confirmed a dust mite allergy and prescribed Montelukast. She remained well during the summer months however during winter she had 3 distinctive episodes of wheeze and cough which were managed by antibiotics and prednisolone.

In the emergency department, an echocardiogram was performed which showed a cardiac tamponade. She was transferred to CICU where she had a pericardial drain inserted. The fluid was abundant with inflammatory cells. Multiple investigations were performed as follows: Hb: 135g/L, wbc: 20.30 x 10 9/L, Eosinophils: 12.77 x 10 9/L, CRP: 51 mg/L, ESR: 75 mm/hr, LDH: 1188 IU/L, IgE: 8000 UI/ml, ANA, ANCA: negative. CT chest showed mediastinal lymphadenopathy and patchy bilateral infiltrate and cardiac MRI showed myopericarditis and LV fibrosis. BMA showed no malignant cells and sinusitis was confirmed by CT.

On examination, she was underweight. Her nasal mucosa looked inflamed. Otherwise systemic examination was unremarkable.

In the context of poor ejection fraction (20%) with LV fibrosis, urgent MDT was arranged and concluded that our working diagnosis was EGPA. The decision was made to start IV methylprednisolone 10mg/kg/day for 3 days and Ivermectin.

That night our patient had a VF arrest which required a single shock conversion 4J/kg. There was 7-minute downtime.

Treatment was escalated to include cyclophosphamide, rituximab and plasmapheresis. The patient made a remarkable recovery, extubated and transferred to a normal ward.

Her eosinophils count and inflammatory markers improved dramatically following treatment. However, she developed severe neuropathic left leg pain and NCS confirmed peripheral neuropathy.

**Discussion/Results:**

EGPA is a very rare disease and diagnosis can be challenging especially with the absence of histopathology diagnosis.

Early empirical treatment especially in a very ill child in intensive care unit can save lives and divert the progress of the disease.

This patient has fulfilled the American College of Rheumatology criteria to diagnose EGPA including asthma, eosinophil count > 10% of upper normal, peripheral neuropathy, pulmonary infiltrates on CT thorax and paranasal sinuses abnormalities. Cardiac biopsy of the fibrotic mass may be a useful tool for diagnosis; however, this invasive procedure may expose this patient with high risk of fatal arrhythmias. Since other causes of eosinophilia were ruled out including parasitic infections, lymphoproliferative disorders, and rare primary immunodeficiency syndromes (hyper-IgE syndrome due to STAT3 or DOCK8 deficiency and Omenn syndrome) and the patient responded well to treatment, the diagnosis of EGPA was supported.

**Key learning points/Conclusion:**

Asthma not responding to bronchodilator could be another diagnosis Eosinophilia should be interpreted with caution.

Defer the need for histopathology diagnosis in critically ill children

Cardiac involvement is a life-threatening marker

Early diagnosis prevents life threatening complications.

